# Hydration-related indicators and adiposity measures in children and adolescents with abdominal obesity: a clinical cross-sectional study

**DOI:** 10.3389/fped.2026.1925985

**Published:** 2026-09-14

**Authors:** Jiaying Cao, Lu Xu, Ying Wu, Qian Wang

**Affiliations:** 1 Pediatric Department, Shanghai General Hospital, Shanghai Jiao Tong University School of Medicine, Shanghai, China; 2 Division of Neonatology, Anhui Province Children’s Hospital, Hefei, China

**Keywords:** abdominal obesity, children and adolescents, estimated plasma osmolality, urine specific gravity, waist-to-height ratio

## Abstract

**Background:**

The relationship between hydration-related markers and abdominal adiposity severity in children with established abdominal obesity remains poorly understood. This study examined the associations of estimated plasma osmolality (ePosm) and urine specific gravity (USG) with waist-to-height ratio (WHtR) and BMI z-score in children and adolescents with abdominal obesity.

**Methods:**

This single-center cross-sectional study included 207 children and adolescents aged 6–17 years with abdominal obesity. WHtR and BMI z-score were used to characterize central and general adiposity, respectively. ePosm was estimated from serum sodium, potassium, glucose, and urea, while USG was obtained from routine urinalysis. Linear regression models were used to evaluate associations of ePosm and USG with WHtR and BMI z-score after adjusting for potential confounders.

**Results:**

The mean BMI was 29.43 ± 5.20 kg/m², mean BMI z-score was 2.44 ± 0.81, and mean WHtR was 0.62 ± 0.06. ePosm was inversely associated with WHtR in the overall cohort (*β* = −0.025 per 10 mOsm/kg, 95% CI: −0.041 to −0.009; *P* = 0.002), and reached statistical significance among boys aged 6–12 years in subgroup analysis. The association between ePosm and BMI z-score was weaker and less consistent. USG was not significantly associated with either WHtR or BMI z-score.

**Conclusions:**

In children and adolescents with abdominal obesity, ePosm was inversely associated with WHtR, whereas USG showed no consistent association with adiposity phenotypes. The subgroup findings should be considered exploratory. Larger prospective studies are needed to confirm these findings.

## 1 Introduction

Overweight and obesity in children and adolescents have become major global public health challenges, with prevalence continuing to rise and excess adiposity emerging at increasingly younger ages and persisting for longer periods across the life course. The global burden of overweight and obesity among children and adolescents has increased substantially between 1990 and 2021 and is projected to continue rising in the coming years (1). Current estimates indicate that 8.5% of children and adolescents worldwide are living with obesity and 14.8% with overweight (2). In China, national survey data have shown that the prevalence of obesity in children and adolescents was 8.25% over the period from 1985 to 2019 (3). Against the backdrop of rapid socioeconomic development, lifestyle transitions, and changing dietary patterns, the prevention and control of childhood obesity remain particularly challenging (4). Obesity in childhood not only increases the risk of metabolic abnormalities, insulin resistance, hypertension, and other cardiovascular risk factors, but also frequently tracks into adulthood, thereby conferring a higher risk of type 2 diabetes, cardiovascular disease, and all-cause mortality later in life (5). Therefore, beyond identifying obesity status alone, further characterization of clinically measurable obesity-related phenotypes may help refine the assessment of established obesity in children and adolescents and provide a basis for future phenotype-informed management strategies.

Body mass index (BMI) remains the most widely used anthropometric indicator for assessing obesity in children and adolescents. However, BMI primarily reflects overall body size and cannot adequately distinguish fat mass from lean mass, nor does it capture patterns of fat distribution (6, 7). Increasing evidence suggests that central adiposity, rather than elevated BMI alone, is more closely associated with adverse metabolic outcomes, including metabolic syndrome, non-alcoholic fatty liver disease, insulin resistance, and cardiovascular risk (8, 9). Accordingly, measures of central obesity, particularly waist-to-height ratio (WHtR), have attracted growing attention. WHtR is simple to obtain, easy to interpret, and less influenced by height differences, making it a valuable marker for identifying abdominal adiposity and related metabolic risk in children and adolescents (10). The combined use of BMI and WHtR may therefore provide a more comprehensive characterization of obesity phenotypes by capturing both overall adiposity and abnormal central fat distribution.

Beyond diet, physical activity, sleep, and genetic susceptibility, hydration status has recently emerged as a potentially relevant and modifiable physiological correlate of adiposity and metabolic health among children and adolescents (11). Hydration status influences not only fluid balance, renal concentrating function, and osmotic regulation, but may also be linked to body weight and fat accumulation through several behavioral and metabolic pathways, including thirst-driven drinking behavior, replacement of water with sugar-sweetened beverages, appetite regulation, energy intake, and metabolic homeostasis (12–14). Previous studies have suggested that inadequate hydration is common in children, with relatively high prevalence reported in both school-aged children and adolescents (15). A 2025 systematic review further showed that inadequate hydration assessed using urinary biomarkers is widespread among children and adolescents regardless of athletic status, indicating that abnormal hydration is not restricted to specific circumstances but may represent a broader and underrecognized issue in routine child health management (16).

Available evidence suggests that poorer hydration status may be associated with higher adiposity and an increased risk of overweight or obesity (17). However, the current literature remains limited and highly heterogeneous with respect to study populations, exposure measures, outcome definitions, and statistical approaches. For example, some recent school-based studies reported there was no consistent association between BMI or WHtR and urinary hydration markers, highlighting the lack of consensus in this field (18). In addition, most prior studies on hydration status and obesity in children and adolescents have focused on BMI, BMI z-score, body fat percentage, or overall body composition, whereas comparatively less attention has been paid to the abdominal obesity phenotype reflected by WHtR (17, 19). In real-world clinical settings, it remains unclear whether estimated plasma osmolality (ePosm) and urine specific gravity (USG)—both readily obtainable from routine clinical testing—show distinct associations with WHtR and BMI z-score among children with abdominal obesity.

In this clinical cross-sectional study, we investigated the associations of two routinely available hydration-related indicators, ePosm and USG, with WHtR and BMI z-score in children and adolescents with established abdominal obesity. We further examined whether these associations differed by adiposity phenotype, hydration marker, age, and sex, aiming to clarify their clinical relevance for phenotyping abdominal obesity in children.

## 2 Methods

### 2.1 Study design

This was a single-center, retrospective, cross-sectional clinical observational study designed to evaluate the associations between hydration-related indicators and obesity phenotypes in children and adolescents with abdominal obesity. Clinically accessible ePosm and USG were used as indicators of hydration status, whereas WHtR and BMI z-score were used to represent the severity of abdominal obesity and overall obesity, respectively. Differences in these associations across age and sex subgroups were further examined.

### 2.2 Study population

The study population comprised all eligible children and adolescents with clinically diagnosed obesity who attended Shanghai General Hospital between January 2020 and March 2026. Given the retrospective design, no formal a prior sample size calculation was performed. During the study period, 358 children and adolescents aged 6–17 years with clinically diagnosed obesity were initially identified, of whom 353 met the criterion for abdominal obesity. Subsequently, 146 records were excluded because of repeated records (*n* = 2), fever (*n* = 2), acute gastroenteritis (*n* = 1), diabetes mellitus (*n* = 5), or missing data on the main study variables (*n* = 136). For participants with multiple eligible records, only the first eligible record was retained. A total of 207 participants were ultimately included in the analysis. The complete participant selection process is presented in Figure 1.

**Figure 1:**
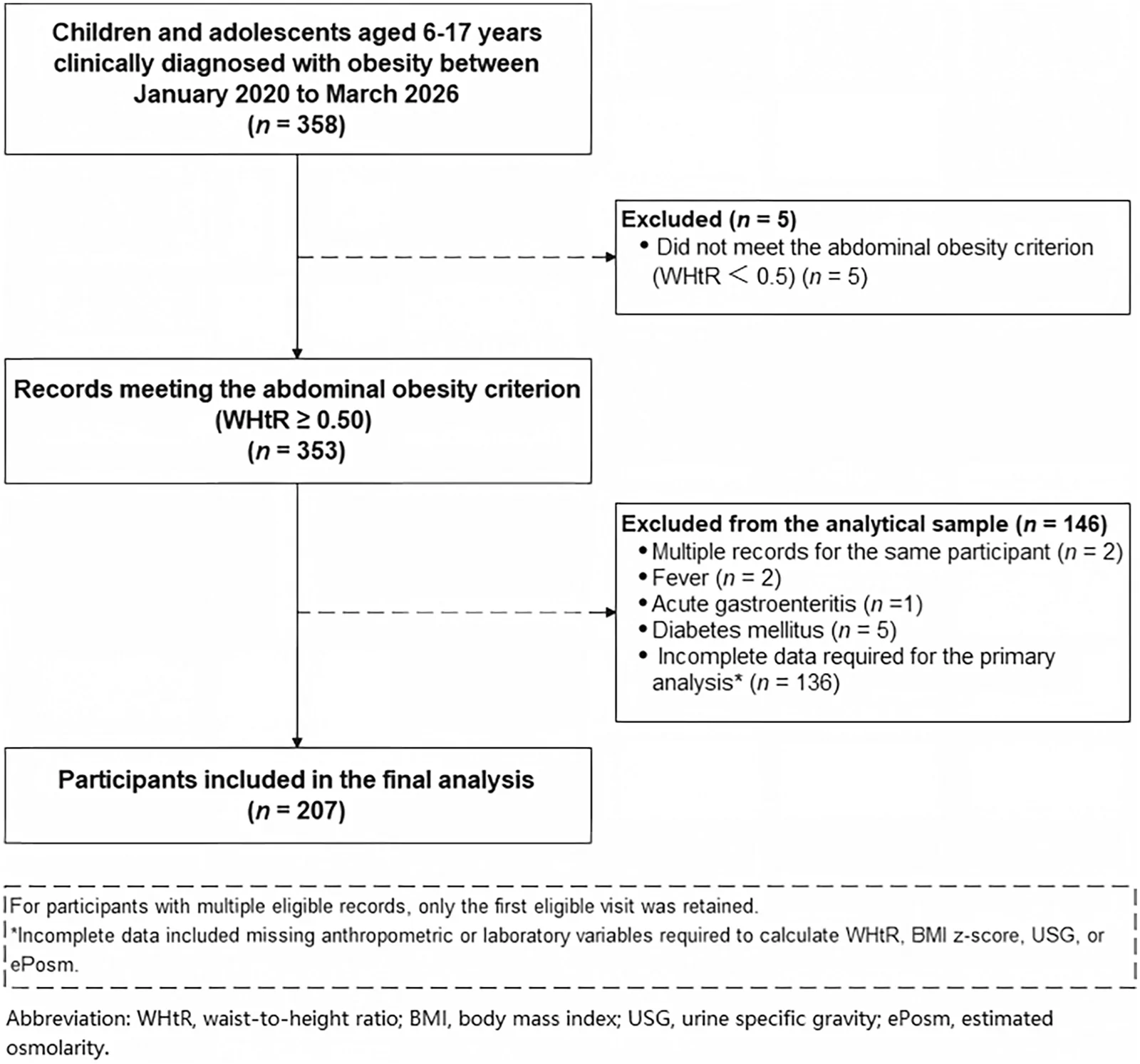
Participant flow diagram.

The inclusion criteria were as follows: age 6–17 years; either sex; fulfillment of the diagnostic criteria for abdominal obesity; completion of height, weight, and waist circumference (WC) measurements during the study period; completion of urinalysis, electrolyte testing, and blood biochemistry; and availability of complete data required to calculate BMI, BMI z-score, WHtR, USG, and ePosm. All anthropometric measurements and blood and urine samples included in the analysis were obtained during the same clinical visit on the day of presentation. Participants were non-fasting at the time of sample collection.

The exclusion criteria were as follows: the presence of diseases that could substantially affect hydration status or the assessment of obesity phenotypes, including chronic kidney disease, confirmed endocrine disorders, and other systemic conditions affecting fluid balance, renal concentrating function, or metabolic status; use within the preceding month of medications that might materially influence hydration status, urinary concentrating function, or body weight metabolism, such as diuretics; acute dehydration, severe infection, acute gastrointestinal illness, or other conditions that could markedly alter fluid status over the short term; inability to complete anthropometric measurements or laboratory testing; and missing data on the main study variables.

### 2.3 Anthropometric measurements and obesity phenotypes

Height, weight, and WC were obtained from routine clinical measurement records during the study period. All personnel responsible for anthropometric assessment underwent standardized theoretical instruction and practical training before performing measurements. Competency was assessed by evaluating intraobserver and interobserver measurement consistency, and only personnel meeting the predefined quality requirements were permitted to perform clinical measurements. Equipment quality control and random measurement audits were conducted periodically to minimize measurement variability and potential measurement bias. Height and weight were measure using a WS-H201 ultrasonic height and weight measurement system (Shanghai Woshen, China), and WC was measured using a Seca 203 non-elastic measuring tape (Seca, Germany). The measurement equipment was calibrated monthly according to routine quality-control procedures. Height, weight, and WC were each measured two times, and the mean of the two measurements was used for analysis.

For height measurement, participants stood barefoot in an upright position with both feet together, with the heels, buttocks, and upper back positioned as close as possible to the stadiometer. The head was maintained in a natural horizontal position, and height was recorded to the nearest 0.1 cm. The repeatability error for height measurements was required to be ≤ 0.2%.

For weight measurement, participants wore light clothing and no shoes, and body weight was measured using a calibrated electronic scale to the nearest 0.1 kg. The difference between repeated weight measurements was required to be ≤ 0.1 kg.

For WC measurement, participants stood naturally with their feet shoulder-width apart, arms relaxed at their sides, the abdomen relaxed, and breathing steady. A non-elastic measuring tape was placed horizontally around the abdomen at the midpoint between the lower margin of the rib cage and the upper border of the iliac crest. The measurement was taken at the end of expiration and recorded to the nearest 0.1 cm. If the difference between repeated WC measurements exceeded 1 cm, the measurements were discarded and the two-measurements procedure were repeated.

BMI was calculated as:

$$BMI=\frac{weight\;(kg)}{height\;{\left(m\right)}^{2}}$$

Given that BMI in children and adolescents varies by age and sex, age- and sex-specific BMI z-scores were further calculated. BMI z-scores were derived using the CDC Extended BMI-for-age Growth Charts for Children and Adolescents data files, with the corresponding parameters matched by sex and age in months. For BMI values at or below the 95th percentile for age and sex, the conventional LMS method was used. For BMI values above the 95th percentile, the CDC extended method incorporating the sigma parameter was applied to calculate the extended BMI z-score, thereby reducing the compression effect of traditional BMI z-scores at very high BMI levels (20).

The calculation was performed as follows:

$$Z=\{\begin{array}{c} \frac{{\left(BMI/M\right)}^{L}-1}{L\times S},BMI\leq P_{95}\\ \Phi^{-1}\left(\frac{90+10\Phi \left(\frac{BMI-P_{95}}{\sigma }\right)}{100}\right),BMI>P_{95} \end{array}$$

WHtR was calculated as:

$$WHtR=\frac{WC\;(cm)}{height\;(cm)}$$

Abdominal obesity was defined as WHtR ≥0.50. This cut-off was selected because it is a simple and widely used screening threshold for increased abdominal adiposity in children and adolescents. It is supported by current international guidance, and has been applied in large pediatric populations, including Chinese children and adolescents. It is also consistent with the practical public health message that WC should be maintained at less than half of height (21–23). In subsequent analyses, WHtR was treated as a continuous variable to reflect the severity of abdominal obesity.

### 2.4 Hydration-related indicators

ePosm and USG were used as the primary indicators of hydration status in this study. USG was obtained from routine urinalysis results and used as an indicator of urinary concentration. USG was measured in random urine samples using a refractometric method on a CM-4500 Fully Automatic Urinalysis System (Medicside, China). Before routine testing. The specific-gravity function was calibrated using a specific-gravity standard solution of 1.040 and distilled water with a specific gravity of 1.000 to establish a liner calibration relationship. During sample analysis, USG was calculated from the measured A/D value using the two calibration points and the instrument’s liner scaling algorithm. The analytical range for USG was 1.000–1.053, with an accuracy within±0.002. A USG value >1.020 was defined as concentrated urine or potential underhydration.

Serum sodium and potassium were measured by indirect ion-selective electrode potentiometry, glucose by the hexokinase method, and urea by the urease-glutamate dehydrogenase method using a Siemens ADVIA Chemistry XPT analyzer. ePosm was estimated from these routinely measured parameters using the following formula:

$$ePosm=2\times (Na^{+}+K^{+})+glucose+urea$$

Sodium, potassium, glucose, and urea were all expressed in mmol/L (24). ePosm ≥295 mOsm/kg was defined as potential underhydration (25).

### 2.5 Age and sex stratification

As children and adolescents are in dynamic stages of growth and development, age and sex may influence body fat distribution, hydration status, and the relationship between them. Given the limited availability of other potential confounders in the retrospective clinical records, the regression models were considered minimally adjusted. Accordingly, prespecified exploratory stratified analyses by age and sex were performed to examine potential heterogeneity in the observed associations. Age was categorized as 6–12 years and 13–17 years, and sex was categorized as boys and girls. Four combined age–sex subgroups were further defined: boys aged 6–12 years, girls aged 6–12 years, boys aged 13–17 years, and girls aged 13–17 years. Potential effect modification by sex and age group was assessed by including ePosm × sex and ePosm × age-group interaction terms in the regression models.

### 2.6 Exposure variables, adiposity phenotypes, and covariates

The main exposure variables were ePosm and USG, which were used as clinically available hydration-related indicators. Both ePosm and USG were analyzed as continuous variables in the regression models. ePosm was additionally categorized using the cutoff of 295 mOsm/kg for group-based distribution comparisons and graphical presentation.

The adiposity phenotype variables were WHtR and BMI z-score. WHtR was used as the primary indicator of abdominal adiposity severity because all participants had established abdominal obesity, whereas BMI z-score was used as a secondary indicator reflecting overall adiposity relative to age and sex. WHtR and BMI z-score were treated as dependent variables in the regression analyses.

Age and sex were considered the main covariates. Covariates were selected based on biological plausibility and their potential confounding roles. The individual components of ePosm were not additionally included as covariates because they constitute the exposure itself. In the overall models, associations were adjusted for age and sex. In age–sex subgroup analyses, models were adjusted for age.

### 2.7 Statistical analysis

All statistical analyses were performed using R software, version 4.5.2. Continuous variables were presented as mean ± standard deviation or median (interquartile range), as appropriate according to their distribution. Categorical variables were expressed as counts and percentages.

Between-group comparisons for continuous variables were conducted using *t* test, analysis of variance, Mann–Whitney *U* test, or Kruskal—Wallis test, as appropriate. Categorical variables were compared using the chi-square test or Fisher’s exact test.

Linear regression models were used to evaluate the associations of ePosm and USG with WHtR and BMI z-score. The overall models were adjusted for age and sex, whereas the age–sex subgroup models were adjusted for age. Effect estimates for ePosm were expressed per 10 mOsm/kg increase, and effect estimates for USG were expressed per 0.010-unit increase. Results were reported as regression coefficients (*β*), 95% confidence intervals (CIs), and *P* values. The association between ePosm and WHtR in the overall population was considered the primary analysis. The remaining analyses were considered secondary or exploratory. In total, twenty regression comparisons and four additional ePosm-category comparisons were performed. Regression assumptions were evaluated using residual and influence diagnostics. Sensitivity analyses were performed using HC3 heteroscedasticity- robust standard errors and robust regression.

Forest plots were used to illustrate the associations between different hydration indicators and obesity phenotypes across age and sex subgroups. Boxplots overlaid with jittered individual data points were used to display the distribution of WHtR according to ePosm. All statistical tests were two-sided. Unadjusted *P* values were reported for secondary and exploratory analyses and interpreted cautiously in the context of multiple comparisons, together with effect estimates and 95% CIs.

### 2.8 Ethics

This study was based on existing clinical data and did not involve any additional examinations or interventions beyond routine clinical care. All data were de-identified before statistical analysis and were used exclusively for the purposes of this study. The study protocol was reviewed and approved by the Ethics Committee of Shanghai General Hospital (approval No.2026KS348). For this retrospective analysis of existing clinical data, the requirement for informed consent could be waived in accordance with the committee’s approval and applicable ethical regulations.

## 3 Results

### 3.1 Clinical characteristics of the study population

A total of 207 children and adolescents with abdominal obesity were included in the analysis. The clinical characteristics of the overall cohort and age–sex subgroups are shown in Table 1. The mean BMI was 29.43 ± 5.2 kg/m², the mean BMI z-score was 2.44 ± 0.81, the mean WC was 95.0 ± 11.5 cm, and the mean WHtR was 0.62 ± 0.06, indicating a pronounced abdominal obesity phenotype in the study population.

**Table 1:** Clinical characteristics of the study population.

Characteristics	Overall	Aged 6–12 years		Aged 13–17 years		*P*-value
		Boys	Girls	Boys	Girls	
Sample size						
Participants, *n* (%)	207 (100)	113 (54.6)	44 (21.3)	28 (13.5)	22 (10.6)	—
Demographic and anthropometric characteristics						
Age, years	10.86 ± 2.28	10.03 ± 1.57	9.56 ± 1.77	13.89 ± 1.07	13.84 ± 1.05	—
Height, cm	152.76 ± 13.83	149.71 ± 11.51	145.53 ± 13.41	169.20 ± 10.33	161.98 ± 7.44	＜0.001
Weight, kg	69.00 (53.85-81.50)	65.00 (53.50–75.00)	55.65 (45.53–74.70)	92.55 (80.03–99.93)	88.60 (74.72–96.88)	＜0.001
BMI, kg/m^2^	29.43 ± 5.20	29.07 ± 4.00	27.54 ± 7.49	31.39 ± 3.98	32.58 ± 4.58	＜0.001
BMI z-score	2.44 ± 0.81	2.58 ± 0.78	2.37 ± 1.10	2.15 ± 0.39	2.18 ± 0.50	0.021
WC, cm	95.0 ± 11.5	94.3 ± 10.9	88.5 ± 9.7	102.2 ± 9.5	102.6 ± 11.5	＜0.001
WHtR	0.62 ± 0.06	0.63 ± 0.06	0.61 ± 0.07	0.60 ± 0.04	0.63 ± 0.07	0.076
Hydration markers						
USG	1.023 ± 0.006	1.022 ± 0.007	1.024 ± 0.006	1.024 ± 0.006	1.021 ± 0.007	0.211
USG >1.020, *n* (%)	115 (55.6)	60 (53.1)	29 (65.9)	17 (60.7)	9 (40.9)	0.221
ePosm, mOsm/kg	295.60 ± 5.21	295.80 ± 6.18	295.60 ± 3.98	295.80 ± 3.14	294.30 ± 3.96	0.653
ePosm≥295mOsm/kg, *n* (%)	113 (54.6)	56 (49.6)	29 (65.9)	18 (64.3)	10 (45.5)	0.156
Electrolyte and biochemical profiles						
Sodium, mmol/L	138.37 ± 2.03	138.37 ± 2.00	138.62 ± 2.16	138.15 ± 2.00	138.10 ± 2.06	0.714
Potassium, mmol/L	4.32 ± 0.48	4.33 ± 0.39	4.38 ± 0.78	4.29 ± 0.30	4.16 ± 0.30	0.392
Glucose, mmol/L	5.61 ± 2.43	5.50 ± 2.29	5.38 ± 2.23	6.38 ± 3.40	5.62 ± 1.94	0.323
Urea, mmol/L	4.63 ± 3.12	4.90 ± 4.10	4.25 ± 1.05	4.56 ± 1.28	4.14 ± 0.86	0.560
Creatinine, umol/L	43.33 ± 15.30	41.82 ± 18.68	39.25 ± 7.15	51.77 ± 9.01	48.53 ± 7.44	0.001

Date are presented as mean ± SD, median (IQR) or *n* (%). *P* values were obtained using one-way analysis of variance or the Kruskal–Wallis test for continuous variables and the chi-square test or Fisher’s exact test for categorical variables. BMI, body mass index; WC, waist circumference; WHtR, waist-to-height ratio; USG, urine specific gravity; ePosm, estimated plasma osmolality; SD, standard deviation; IQR, interquartile range.

After stratification by age and sex, 113 participants were boys aged 6–12 years (54.6%), 44 were girls aged 6–12 years (21.3%), 28 were boys aged 13–17 years (13.5%), and 22 were girls aged 13–17 years (10.6%). BMI, BMI z-score, and WC differed significantly across the age–sex subgroups. In general, adolescents aged 13–17 years had higher BMI and WC, whereas boys aged 6–12 years had relatively higher BMI z-scores. By contrast, WHtR did not differ significantly across subgroups, suggesting that although overall obesity severity and body size varied by age and sex, the degree of abdominal obesity was broadly comparable among subgroups.

The mean USG was 1.023 ± 0.006, and 115 participants (55.6%) had USG >1.020. The mean ePosm was 295.6 ± 5.21 mOsm/kg, and 113 participants (54.6%) had ePosm ≥295 mOsm/kg. No significant differences were observed across age–sex subgroups in USG, the proportion with USG >1.020, ePosm, or the proportion with ePosm ≥295 mOsm/kg. Likewise, major electrolyte and biochemical parameters, including sodium, potassium, glucose, and urea, were comparable across subgroups. Collectively, these findings indicate that although obesity-related phenotypes varied substantially by age and sex, hydration-related indicators were relatively evenly distributed across subgroups.

### 3.2 Marker-specific associations between hydration-related indicators and obesity phenotypes

To further delineate the relationships between hydration-related indicators and obesity phenotypes, we separately examined the associations of ePosm and USG with WHtR and BMI z-score, with additional stratification by age and sex.

ePosm was inversely associated with WHtR. In the overall population, each 10 mOsm/kg increase in ePosm was associated with a 0.025-unit decrease in WHtR (*β* = −0.025, 95% CI: −0.041 to −0.009, *P* = 0.002). In stratified analyses, this association was primarily observed in boys aged 6–12 years (*β* = −0.021, 95% CI: −0.039 to −0.003, *P* = 0.026), but not in the other subgroups. Formal interaction tests showed no significant effect modification by sex (*P* for interaction=0.694) or age group (*P* for interaction=0.158). Among girls aged 13–17 years, the association was inverse but not statistically significant(*β* = −0.058, 95% CI: −0.132 to 0.015, *P* = 0.112) (Figure 2A). For the primary overall ePosm–WHtR model, the Breusch–Pagan test showed no evidence of heteroscedasticity (*P* = 0.406). Sensitivity analyses yielded consistent results, with the inverse association remaining significant using HC3 robust standard errors (*β* = −0.025, 95% CI: −0.041 to −0.010, *P* = 0.001) and robust regression (*β* = −0.022, 95% CI: −0.034 to −0.009, *P* < 0.001).

**Figure 2:**
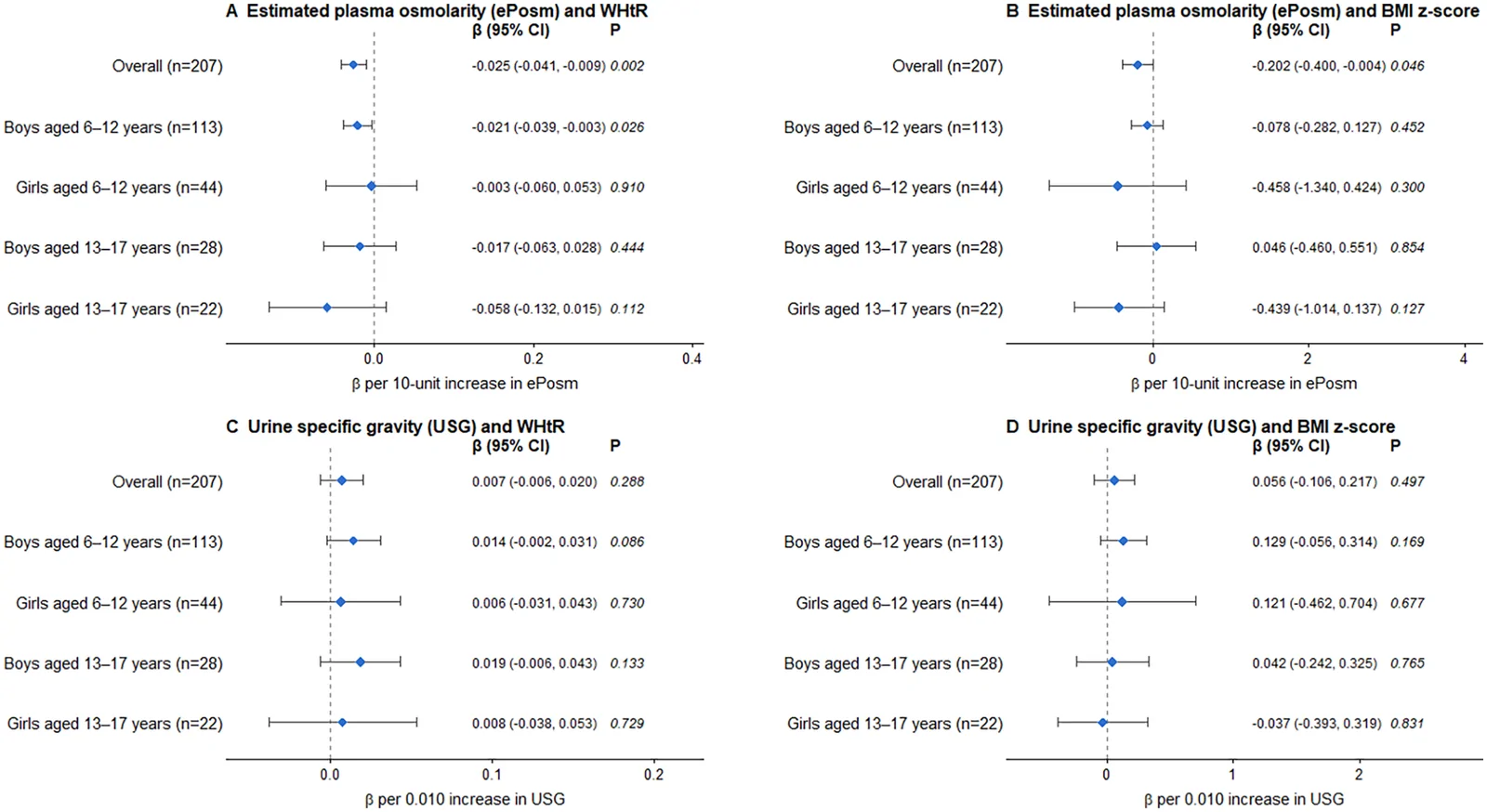
Associations of hydration-related indicators with adiposity measures in the overall population and age-sex subgroups. **(A)** Estimated plasma osmolarity (ePosm) and waist-to-height ratio (WHtR); **(B)** ePosm and BMI z-score; **(C)** Urine specific gravity (USG) and WHtR; **(D)** USG and BMI z-score. Diamonds represent regression coefficients (*β*), and horizontal lines represent 95% confidence intervals (CIs). Effect estimates for ePosm are expressed per 10-unit increase, whereas those for USG are expressed per 0.010-unit increase. Overall models were adjusted for age and sex, whereas age-sex subgroup models were adjusted for age only. The *P* values displayed beside the estimates correspond to the associations within the overall population or individual subgroups. Formal interaction tests showed no significant effect modification by sex or age group for ePosm–WHtR (*P* for interaction=0.694 and 0.158, respectively), ePosm–BMI z-score (0.141 and 0.660), USG–WHtR (0.229 and 0.556), or USG–BMI z-score (0.559 and 0.437). Subgroup analyses were considered exploratory.

ePosm also showed a weak inverse association with BMI z-score in the overall population (*β* = −0.202, 95% CI: −0.400 to −0.004, *P* = 0.046). However, this pattern was not consistently reproduced in the stratified analyses, with no significant associations observed in boys aged 6–12 years (*β* = −0.078, 95% CI: −0.282 to 0.127, *P* = 0.452), girls aged 6–12 years (*β* = −0.458, 95% CI: −1.340 to 0.424, *P* = 0.300), boys aged 13–17 years (*β* = 0.046, 95% CI: −0.460 to 0.551, *P* = 0.854), or girls aged 13–17 years (*β* = −0.439, 95% CI: −1.014 to 0.137, *P* = 0.127) (Figure 2B).

In contrast, USG was not consistently associated with either obesity phenotype. In the overall population, each 0.010-unit increase in USG was not significantly associated with WHtR (*β* = 0.007, 95% CI: −0.006 to 0.020, *P* = 0.288), and no significant associations were observed in any age–sex subgroup (Figure 2C). Similar findings were obtained for BMI z-score. No significant association was identified in the overall population (*β* = 0.056, 95% CI: −0.106 to 0.217, *P* = 0.497), and all subgroup estimates remained non-significant (Figure 2D).

### 3.3 Distribution of WHtR according to ePosm status

To further assess the relationship between ePosm and WHtR, participants were categorized according to ePosm as <295 mOsm/kg or ≥295 mOsm/kg, and WHtR distributions were compared within each age–sex subgroup.

Among boys aged 6–12 years, WHtR was significantly lower in those with ePosm ≥295 mOsm/kg than in those with ePosm <295 mOsm/kg (*P* = 0.012). This finding was consistent with the inverse association observed in the linear regression analysis and further supports that the relationship between ePosm and abdominal obesity severity was primarily concentrated in this subgroup.

In girls aged 6–12 years, boys aged 13–17 years, and girls aged 13–17 years, no statistically significant differences in WHtR distribution were observed between ePosm categories, with *P* values of 0.169, 0.701, and 0.582, respectively. Although directional differences were noted in some subgroups, the distributions showed considerable dispersion and no stable between-group separation was evident (Figure 3).

**Figure 3:**
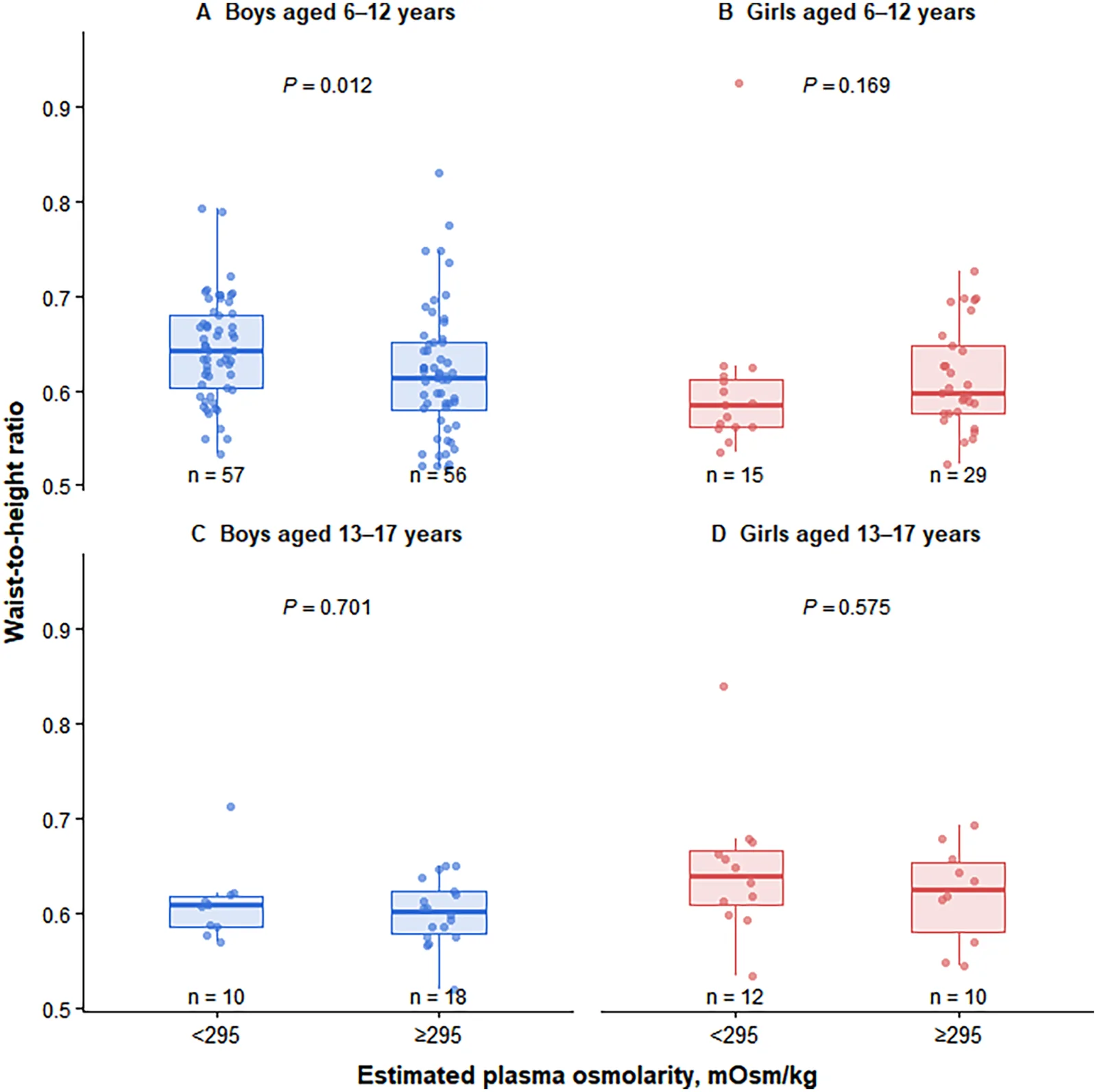
Exploratory comparison of waist-to-height ratio (WHtR) according to estimated plasma osmolarity (ePosm) categories across age-sex subgroups. Participants were categorized according to ePosm as <295 or ≥295. Boxes represent the interquartile range (25th-75th percentiles), the central horizontal line represents the median, and whiskers extend to the most extreme observations within 1.5 times the interquartile range. Jittered points represent individual observations, including observations beyond the whiskers, and the sample size (n) is shown for each category. Between-category comparisons within each age-sex subgroup were performed using the two-sided Wilcoxon rank-sum test (Mann–Whitney U test). The same *y*-axis range was used across all panels. This categorical analysis was exploratory. The primary analysis evaluated ePosm as a continuous variable. **(A)** Boys aged 6–12 years; **(B)** Girls aged 6–12 years; **(C)** Boys aged 13–17 years; **(D)** Girls aged 13–17 years.

## 4 Discussion

Previous pediatric research has focused predominantly on urinary hydration markers, particularly urine osmolality. Several studies have reported that greater obesity is associated with higher urine osmolality, more concentrated urine, and poorer hydration, suggesting that children with obesity may be more susceptible to inadequate hydration (17, 26). However, other studies have found no significant associations between USG and body fat percentage or other body-composition measures, indicating that the available evidence remains inconsistent (27). Unlike studies that primarily used urinary markers and focused on general obesity or body composition, the present study evaluated ePosm and USG as hydration-related indicators and examined their associations with both WHtR and BMI z-score. In this context, our study identified a specific clinical pattern in children and adolescents with abdominal obesity. ePosm was significantly and inversely associated with WHtR, and this association was primarily observed in boys aged 6–12 years. By contrast, the association between ePosm and BMI z-score was weaker, whereas USG showed no stable association with either WHtR or BMI z-score.

The most notable finding of this study was that higher ePosm was associated with lower WHtR. This observation does not fully align with the intuitive assumption that poorer hydration is associated with greater obesity and therefore should not be simplistically interpreted as indicating that more severe dehydration corresponds to more severe abdominal obesity. Rather, one possible interpretation is that, in children with established abdominal obesity, ePosm may reflect a more complex state of fluid regulation rather than merely insufficient water intake. ePosm is determined jointly by sodium, potassium, glucose, urea, and body water status, and is tightly regulated through mechanisms involving thirst, antidiuretic hormone release, and renal water reabsorption. Compared with urinary markers, ePosm more closely reflects systemic osmotic homeostasis of the internal fluid milieu (28, 29). Accordingly, the finding that lower ePosm was accompanied by higher WHtR in children with abdominal obesity may reflect alterations in fluid distribution, relative volume expansion, or adaptive changes in osmotic regulation associated with increasing central adiposity, rather than indicating a more favorable hydration state. However, these proposed explanations remain speculative because fluid distribution, volume status, and osmotic regulatory mechanisms were not directly assessed in this study. This interpretation is broadly consistent with recent research on childhood obesity and body water composition. Obesity in children is not characterized solely by increased fat mass, but is also commonly accompanied by changes in fat-free mass, total body water proportion, and fluid distribution (30, 31). From this perspective, the inverse ePosm–WHtR relationship observed in the present study may not represent a conventional “signal of inadequate drinking,” but may instead provide a preliminary observational signal of possible differences in fluid composition and osmotic regulation across degrees of abdominal obesity severity. In this context, ePosm may represent a promising marker of hydration-related differences associated with abdominal adiposity. This hypothesis requires confirmation in prospective studies using direct and repeated assessments of hydration and body composition.

The stronger association of ePosm with WHtR than with BMI z-score further supports the importance of phenotype selection. Although BMI z-score is widely used for diagnosing and grading obesity in children, it primarily reflects overall deviation in body weight relative to age, sex, and height, and cannot distinguish fat mass, lean mass, body water content, or fat distribution. In children with established abdominal obesity, changes in BMI z-score may therefore fail to accurately capture the severity of the abdominal anthropometric phenotype (32). By contrast, WHtR more directly reflects abdominal fat distribution and central body shape. Recent longitudinal evidence using dual-energy x-ray absorptiometry (DEXA)-derived fat mass as the reference has shown that WHtR has better long-term concordance than BMI with total and trunk fat mass in children (33). Thus, our findings should not be interpreted as showing a general association between ePosm and obesity, but more precisely as indicating that ePosm is associated with the severity of central obesity, with WHtR serving as a more appropriate phenotypic marker of this relationship than BMI z-score.

In this study, USG was not significantly associated with either WHtR or BMI z-score. This finding does not necessarily indicate that hydration status is unrelated to adiposity phenotypes; rather, it highlights that different hydration-related markers may capture different physiological dimensions. USG is a simple and readily available index of urinary concentration, but a single measurement can be influenced by recent fluid intake, timing of urine collection, urine volume, urinary solute load, protein, glucose, and renal concentrating function. It therefore primarily reflects the immediate renal excretory response and may not reliably represent systemic osmotic regulation (34, 35). The observation that ePosm, but not USG, was associated with WHtR suggests that abdominal adiposity severity in children with abdominal obesity may be linked more closely to systemic osmotic fluid regulation than to short-term urinary concentration. This marker specificity is an important finding of the present study and indicates that USG and ePosm should not be treated as interchangeable hydration indicators in future research.

In the present study, the ePosm–WHtR association reached statistical significance in the subgroup of boys aged 6–12 years and was not statistically significant in girls or in adolescents aged 13–17 years. However, formal interaction tests did not provide evidence of effect modification by sex or age group, indicating that differences in statistical significance across subgroups should not be interpreted as evidence of true between-group differences. Children aged 6–12 years are typically in the prepubertal or early pubertal period, during which height growth, fat distribution, increases in lean mass, and body water proportion remain dynamically changing (36, 37). Such developmental changes could potentially contribute to heterogeneity in the observed associations, although this possibility could not be directly evaluated because pubertal stage was unavailable. After the onset of puberty, changes in sex hormones, muscle mass, fat distribution, and sodium–water regulation may also influence the relationship between hydration-related indicators and adiposity (12, 38). At the same time, the boys aged 6–12 years constituted the largest subgroup, whereas the adolescent subgroups were relatively small, resulting in differences in statistical precision across subgroups; therefore, the absence of a statistically significant association in adolescents should not be interpreted as definitive evidence that no such relationship exists. Accordingly, the statistically significant finding among boys aged 6–12 years should be regarded as exploratory and hypothesis-generating rather than evidence of an age- or sex-specific association. Larger studies incorporating pubertal staging, body composition data, and drinking behavior are needed to further examine this possibility.

The relationship between ePosm and the severity of abdominal obesity may involve the arginine vasopressin system. Arginine vasopressin is a key hormone in the regulation of osmotic homeostasis and water–electrolyte balance, and its secretion is highly sensitive to changes in ePosm. Copeptin, a stable surrogate marker of vasopressin, has increasingly been proposed as a link between hydration status, osmotic regulation, and metabolic abnormalities. Pediatric studies have shown that water deprivation increases serum sodium, ePosm, and copeptin levels. Meanwhile, children with obesity have been reported to have higher copeptin concentrations than their non-obese peers, suggesting that obesity may be accompanied by alterations in the vasopressin axis (39). Studies in adults have likewise shown that higher water intake, lower urine osmolality, and lower vasopressin secretion are associated with more favorable metabolic profiles, supporting the possibility of an endocrine pathway linking hydration status to metabolic risk (40). However, it should be emphasized that copeptin and vasopressin were not measured in the present study, and this mechanism therefore cannot be directly demonstrated. Accordingly, a potential “osmotic regulation–vasopressin axis–central adiposity phenotype” pathway should be regarded as a hypothesis for future investigation rather than a mechanism supported directly by the present data. This perspective also helps explain why our results did not simply show that higher ePosm was associated with higher WHtR. In real-world clinical populations, ePosm is likely influenced simultaneously by water intake, solute load, renal regulation, insulin status, fluid distribution, and neuroendocrine feedback (28, 29, 41). Thus, the lower ePosm observed in children with higher WHtR may reflect differences in fluid or osmotic regulation.

The main strengths of this study are that it was conducted in a real-world clinical setting and focused specifically on children with abdominal obesity, a clinically meaningful population with clear metabolic risk. The relatively large sample size for a pediatric clinical cohort strengthened the precision and robustness of the overall analyses. Unlike studies relying solely on BMI to characterize obesity, the present study used WHtR as a core phenotypic indicator, thereby providing a more direct representation of abdominal adiposity severity. In addition, we compared two clinically accessible hydration-related indicators, ePosm and USG, and evaluated them in relation to two obesity phenotypes, WHtR and BMI z-score. The use of multivariable regression models and age- and sex-specific subgroup analyses allowed potential heterogeneity in these associations to be examined in greater detail. This design enabled us to reveal both marker specificity and phenotype specificity in the relationship between hydration status and obesity phenotype. Several limitations should also be acknowledged. First, this was a retrospective cross-sectional analysis conducted at a single center, and the causal direction between ePosm and WHtR cannot be determined. In addition, center-specific referral patterns, clinical practices, and population characteristics may limit the generalizability of the finding. Second, all participants had abdominal obesity, and no comparison group of children without abdominal obesity or with normal weight was included. The findings are therefore more suitable for interpreting phenotypic variation within abdominal obesity and cannot be directly generalized to the broader population of children and adolescents. Third, ePosm was calculated from routinely measured serum sodium, potassium, glucose, and urea concentrations rather than directly measured. ePosm may not fully capture unmeasured osmotically active solutes, and may be affected by the choice of calculation formula, laboratory measurement error, and short-term variations in fluid intake, diet, or physiological status. In addition, both ePosm and USG were derived from single-time-point samples. A single USG measurement may be influenced by recent fluid and food intake, the timing of urine collection, urinary solute load, glycosuria or proteinuria, and renal concentrating function. Consequently, measurement error and exposure misclassification cannot be excluded, and neither ePosm nor a single USG measurement can fully represent long-term hydration status. Fourth, the regression models were minimally adjusted for age and sex, and residual confounding cannot be excluded. Detailed information on total water and fluid intake, beverage type, dietary sodium and protein intake, simple-carbohydrate and total energy intake, overall diet quality, 24-hour urine volume, renal function or renal concentrating capacity, physical activity and sweating, season and ambient temperature, supplement and medication use, socioeconomic conditions, and access to safe drinking water was not systematically available. Pubertal stage, body composition, and copeptin were also unavailable. These factors may influence both hydration-related indicators and adiposity measures and may therefore have affected the observed associations. Fifth, pubertal stage was not assessed in this study. Because pubertal maturation is accompanied by substantial changes in sex hormones, body composition, fat distribution, and fluid regulation, the absence of pubertal staging limited our ability to distinguish the effects of chronological age from those of biological maturation. Finally, some age–sex subgroups, particularly those aged 13–17 years, had relatively small sample sizes, which may have reduced statistical power and limited the ability to detect meaningful associations. The corresponding stratified findings should therefore be considered exploratory and require confirmation in larger and prospective studies.

## 5 Conclusion

In children and adolescents with abdominal obesity, ePosm was inversely associated with WHtR. Although the association reached statistical significance in boys aged 6–12 years in subgroup analysis, formal interaction tests did not support significant effect modification by age or sex, and this subgroup finding should be considered exploratory. These findings do not establish causality or diagnostic utility. Prospective studies are needed to confirm this association and clarify its underlying physiological mechanisms.

## Data Availability

The datasets generated and/or analyzed during the current study are not publicly available because they contain clinical information from pediatric patients, but de-identified data may be available from the corresponding author upon reasonable request and with permission from the Ethics Committee of Shanghai General Hospital.

## References

[1] Kerr JA Patton GC Cini KI Abate YH Abbas N Abd Al Magied AHA. Global, regional, and national prevalence of child and adolescent overweight and obesity, 1990–2021, with forecasts to 2050: a forecasting study for the global burden of disease study 2021. Lancet. (2025) 405:785–812. 10.1016/S0140-6736(25)00397-6 40049185 PMC11920006

[2] Zhang X Liu J Ni Y Yi C Fang Y Ning Q. Global prevalence of overweight and obesity in children and adolescents: a systematic review and meta-analysis. JAMA Pediatr. (2024) 178(8):800. 10.1001/jamapediatrics.2024.1576 38856986 PMC11165417

[3] Song X Zhou B Baird S Lu C Ezzati M Chen L. Trends and inequalities in thinness and obesity among Chinese children and adolescents: evidence from seven national school surveys between 1985 and 2019. Lancet Public Health. (2024) 9(12):e1025–36. 10.1016/S2468-2667(24)00211-1 39481418 PMC7616785

[4] Ball GDC Merdad R Birken CS Cohen TR Goodman B Hadjiyannakis S. Managing obesity in children: a clinical practice guideline. CMAJ. (2025) 197(14):E372–89. 10.1503/cmaj.241456 40228835 PMC12007946

[5] Larruy-García A Mahmood L Miguel-Berges ML Masip G Seral-Cortés M De Miguel-Etayo P. Diet quality scores, obesity and metabolic syndrome in children and adolescents: a systematic review and meta-analysis. Curr Obes Rep. (2024) 13(4):755–88. 10.1007/s13679-024-00589-6 PMC11522196 39331350

[6] Hampl SE Hassink SG Skinner AC Armstrong SC Barlow SE Bolling CF. Clinical practice guideline for the evaluation and treatment of children and adolescents with obesity. Pediatrics. (2023) 151(2):e2022060640. 10.1542/peds.2022-060640 36622115

[7] Sweatt K Garvey WT Martins C. Martins C strengths and limitations of BMI in the diagnosis of obesity: what is the path forward? Curr Obes Rep. (2024) 13(3):584–95. 10.1007/s13679-024-00580-1 38958869 PMC11306271

[8] Marketou ME Buechler NS Fragkiadakis K Plevritaki A Zervakis S Maragkoudakis S. Visceral fat and cardiometabolic future in children and adolescents: a critical update. Pediatr Res. (2023) 94(5):1639–47. 10.1038/s41390-023-02709-9 37402844

[9] Ross R Neeland IJ Yamashita S Shai I Seidell J Magni P. Waist circumference as a vital sign in clinical practice: a consensus statement from the IAS and ICCR working group on visceral obesity. Nat Rev Endocrinol. (2020) 16(3):177–89. 10.1038/s41574-019-0310-7 32020062 PMC7027970

[10] Lo K Wong M Khalechelvam P Tam W. Waist-to-height ratio, body mass index and waist circumference for screening paediatric cardio-metabolic risk factors: a meta-analysis. Obes Rev. (2016) 17(12):1258–75. 10.1111/obr.12456 27452904

[11] García AIL Moráis-Moreno C Samaniego-Vaesken ML Puga AM Varela-Moreiras G Partearroyo T. Association between hydration Status and body composition in healthy adolescents from Spain. Nutrients. (2019) 11(11):2692. 10.3390/nu11112692 PMC6893474 31703309

[12] Zborowski M Skotnicka M. The role of hydration in children and adolescents—a theoretical framework for reviewing recommendations, models, and empirical studies. Nutrients. (2025) 17(17):2841. 10.3390/nu17172841 40944233 PMC12430526

[13] Calcaterra V Cena H Magenes VC Vincenti A Comola G Beretta A. Sugar-Sweetened beverages and metabolic risk in children and adolescents with obesity. A Narrative Review. Nutrients. (2023) 15(3):702. 10.3390/nu15030702 PMC9918944 36771409

[14] Muckelbauer R Barbosa CL Mittag T Burkhardt K Mikelaishvili N Müller-Nordhorn J. Association between water consumption and body weight outcomes in children and adolescents: a systematic review. Obesity (Silver Spring). (2014) 22(12):2462–75. 10.1002/oby.20911 25401930

[15] Iglesia I Guelinckx I De Miguel-Etayo PM González-Gil EM Salas-Salvadó J Kavouras SA. Total fluid intake of children and adolescents: cross-sectional surveys in 13 countries worldwide. Eur J Nutr. (2015) 54(S2):57–67. 10.1007/s00394-015-0946-6 26081646 PMC4473088

[16] Papaoikonomou G Apergi K Malisova O. Children, adolescents and urine hydration indices—a systematic literature review on athletes and non-athletes. Children (Basel). (2025) 12(2):171. 10.3390/children12020171 40003272 PMC11854905

[17] Kozioł-Kozakowska A Wójcik M Stochel-Gaudyn A Szczudlik E Suder A Piórecka B. The severity of obesity promotes greater dehydration in children: preliminary results. Nutrients. (2022) 14(23):5150. 10.3390/nu14235150 PMC9739486 36501180

[18] Piórecka B Drapała N Kozioł-Kozakowska A Jagielski P Płonka M Kawalec P. Prevalence of underhydration among schoolchildren and their mothers from the city and municipality of niepolomice and cracow in southern Poland. J Physiol Pharmacol. (2025) 76(2):147–54. 10.26402/jpp.2025.2.04 40350648

[19] Maffeis C Tommasi M Tomasselli F Spinelli J Fornari E Scattolo N. Fluid intake and hydration status in obese vs normal weight children. Eur J Clin Nutr. (2016) 70(5):560–5. 10.1038/ejcn.2015.170 26463726

[20] Freedman DS Davies AJG Kompaniyets L Lange SJ Goodman AB Phan T-LT. A longitudinal comparison of alternatives to body mass Index Z-scores for children with very high body mass indexes. J Pediatr. (2021) 235:156–62. 10.1016/j.jpeds.2021.02.072 33676932

[21] Mulvihill C Parretti HM Aswani N Committee G. Overweight and obesity management: summary of updated NICE guidance. Br Med J. (2025) 391:r2286. 10.1136/bmj.r2286 41326143

[22] Zhang Y-X Chen J Liu X-H. Screening of central obesity among normal-weight children and adolescents in shandong, China. Br J Nutr. (2021) 126(6):950–5. 10.1017/S0007114520004882 33292920

[23] Ashwell M Gibson S. A proposal for a primary screening tool: `keep your waist circumference to less than half your height’. BMC Med. (2014) 12(1):207. 10.1186/s12916-014-0207-1 25377944 PMC4223160

[24] Martín-Calderón JL Bustos F Tuesta-Reina LR Varona JM Caballero L Solano F. Choice of the best equation for plasma osmolality calculation: comparison of fourteen formulae. Clin Biochem. (2015) 48(7–8):529–33. 10.1016/j.clinbiochem.2015.03.005 25794428

[25] Liska D Mah E Brisbois T Barrios PL Baker LB Spriet LL. Narrative review of hydration and selected health outcomes in the general population. Nutrients. (2019) 11(1):70. 10.3390/nu11010070 30609670 PMC6356561

[26] Michels N Van den Bussche K Vande Walle J De Henauw S. Belgian Primary school children’s hydration status at school and its personal determinants. Eur J Nutr. (2017) 56(2):793–805. 10.1007/s00394-015-1126-4 26676743

[27] Clayton P Trak-Fellermeier MA Macchi A Galván R Bursac Z Huffman-Ercanli F. The association between hydration status and body composition in healthy children and adolescents. J Pediatr Endocrinol Metab. (2023) 36(5):470–7. 10.1515/jpem-2022-0462 37006164 PMC10174625

[28] Dmitrieva NI Boehm M Yancey PH Enhörning S. Long-term health outcomes associated with hydration status. Nat Rev Nephrol. (2024) 20(5):275–94. 10.1038/s41581-024-00817-1 38409366 PMC13592372

[29] Bichet DG. Regulation of thirst and vasopressin release. Annu Rev Physiol. (2019) 81(1):359–73. 10.1146/annurev-physiol-020518-114556 30742785

[30] Zhang J Cao W Xu J Wang H Luo R Gan Q. Overweight and obese children aged 6–17 years in China had lower level of hydration Status: a cross-sectional study. Nutrients. (2025) 17(2):364. 10.3390/nu17020364 39861494 PMC11768175

[31] Celik A Cebeci AN. Evaluation of hydration status of children with obesity—a pilot study. J Pediatr Endocrinol Metab. (2021) 34(3):295–300. 10.1515/jpem-2020-0495 33675213

[32] Vanderwall C Eickhoff J Randall Clark R Carrel AL. BMI z-score in obese children is a poor predictor of adiposity changes over time. BMC Pediatr. (2018) 18(1):187. 10.1186/s12887-018-1160-5 29880034 PMC5992682

[33] Agbaje AO. Waist-circumference-to-height-ratio had better longitudinal agreement with DEXA-measured fat mass than BMI in 7237 children. Pediatr Res. (2024) 96(5):1369–80. 10.1038/s41390-024-03112-8 38443520 PMC11522001

[34] Barley OR Chapman DW Abbiss CR. Reviewing the current methods of assessing hydration in athletes. J Int Soc Sports Nutr. (2020) 17(1):52. 10.1186/s12970-020-00381-6 33126891 PMC7602338

[35] Suh HG Summers LDG Seal AD Colburn AT Mauromoustakos A Perrier ET. Afternoon urine osmolality is equivalent to 24 h for hydration assessment in healthy children. Eur J Clin Nutr. (2020) 74(6):884–90. 10.1038/s41430-019-0519-5 31624367 PMC7283040

[36] Zhao Y Gong J-X Ji Y-T Zhao X-Y He L Cai S-Z. Cross-sectional study of characteristics of body composition of 24,845 children and adolescents aged 3–17 years in suzhou. BMC Pediatr. (2023) 23(1):358. 10.1186/s12887-023-04134-7 37442965 PMC10339479

[37] Chun D Kim SJ Suh J Kim J. Big data-based reference centiles for body composition in Korean children and adolescents: a cross-sectional study. BMC Pediatr. (2024) 24(1):692. 10.1186/s12887-024-05166-3 39478496 PMC11523643

[38] Cuda S Censani M O'Hara V Paisley J Kharofa R Conroy R. Special considerations for the child with obesity: an obesity medicine association (OMA) clinical practice statement (CPS) 2024. Obes Pillars. (2024) 11:100113. 10.1016/j.obpill.2024.100113 38953014 PMC11216014

[39] Tuli G Munarin J Tessaris D Einaudi S Matarazzo P De Sanctis L. Distribution of plasma copeptin levels and influence of obesity in children and adolescents. Eur J Pediatr. (2021) 180(1):119–26. 10.1007/s00431-020-03777-3 32809080 PMC7782451

[40] Brunkwall L Ericson U Nilsson PM Enhörning S. High water intake and low urine osmolality are associated with favorable metabolic profile at a population level: low vasopressin secretion as a possible explanation. Eur J Nutr. (2020) 59(8):3715–22. 10.1007/s00394-020-02202-7 32072267 PMC7669756

[41] Gizowski C Bourque CW. The neural basis of homeostatic and anticipatory thirst. Nat Rev Nephrol. (2018) 14(1):11–25. 10.1038/nrneph.2017.149 29129925

